# Dietary resveratrol modulates rumen microbiota and metabolic function in Tibetan sheep: an integrated 16S rRNA sequencing and metabolomics analysis

**DOI:** 10.3389/fmicb.2025.1679856

**Published:** 2025-12-12

**Authors:** Wenling Xie, Yu Zhang, Yuan Feng, Yujie Li, Linsheng Gui, Shengzhen Hou, Lijuan Han, Zhenzhen Yuan, Chao Yang, Shengnan Sun

**Affiliations:** 1 College of Agriculture and Animal Husbandry, Qinghai University, Xining, Qinghai, China; 2 Department of Pharmacy, College of Medicine, Qinghai University, Xining, China; 3 State Key Laboratory of Plateau Ecology and Agriculture, Qinghai University, Xining, China

**Keywords:** Tibetan sheep, rumen development, 16S rRNA, resveratrol, microbiota, metabolites

## Abstract

**Introduction:**

Resveratrol (RES), a natural plant polyphenolic compound, can be used as a dietary supplement and has shown good application after addition to monogastric animal diets, but fewer studies have been conducted in ruminants, especially in Tibetan sheep where no deeper studies have been made. In this study, we used 16S rRNA and metabolomics systems to investigate the effects of different doses of resveratrol added to the diets of Tibetan sheep on the microbial community structure and metabolomics of their rumen.

**Results:**

The results showed a significant (*p* < 0.05) increase in rumen papilla length in the H-RES group, along with elevated acetate and butyrate concentrations. Microbial analysis revealed significantly higher (*p* < 0.05) relative abundance of *Firmicutes* in the H-RES group, including the genera *Prevotella* and *Ruminococcus*. Metabolic pathway enrichment analysis revealed significant (*p* < 0.05) enrichment of amino acid metabolism-related pathways. In addition, specific microbial genera, *Lactobacillus* spp. and *Ruminococcus* spp. showed significant correlations with metabolites such as pantothenic acid and isoleucine, indicating differential regulatory effects.

**Conclusion:**

In summary, daily supplementation with 1.5 g of resveratrol (H-RES) improved rumen morphology and fermentation processes in Tibetan sheep. These improvements were closely related to changes in microbial community structure and metabolite interactions. The study of rumen microbial community structure and metabolite changes provides new ideas for regulating the rumen environment of Tibetan sheep.

## 1 Introduction

Tibetan sheep (*Ovis aries*) are an endemic germplasm resource unique to the Tibetan Plateau. They constitute a primary economic resource for Tibetan herders and play a vital role in maintaining ecosystem balance (Yan et al., 2024). The rumen is an important component of the digestive system of ruminants, where specific vitamins and enzymes break down feeds into starch and cellulose (Zhang et al., 2021), and they also synthesize the required proteins (Chen et al., 2024) and vitamins (Jing et al., 2020) required for the animal. Rumen microbiota are fundamental to digestive function, supplying up to 70% of the host’s energy and 60% of its amino acids (Bergman, 1990). Small changes in rumen metabolites can affect the rumen microbial environment (Newbold and Ramos-Morales, 2020), so analysis of the microbial community in rumen fluid can provide a scientific basis for determining the feed efficiency (Khiaosa-ard and Zebeli, 2014), nutrient intake (Pereira et al., 2008), growth performance, and meat quality of Tibetan sheep (Shen et al., 2022). Given that the rumen microbiota plays an important role in the energy metabolism and health of Tibetan sheep, it is important to search for natural additives that can modulate their composition and function.

Resveratrol (RES), a natural polyphenolic compound, exhibits defensive properties against fungal and microbial pathogens (Truong et al., 2018) and possesses pharmacological activities, including anti-inflammatory, antioxidant, and cytoprotective effects (Truong et al., 2018). The evidence indicates that RES modulates rumen microbial communities and fermentation processes in ruminants. For instance, dietary RES supplementation increased the abundance of *Prevotella* in pregnant and lactating ewes, improving reproductive health via metabolite and microbiota regulation (Li et al., 2024). In fattening goats, RES significantly reduced the abundance of *Acetitomaculum* and *Moryella*, while enriching taxa involved in short-chain fatty acid production-changes associated with improved growth performance and meat quality (Shen et al., 2022). Preliminary studies in Tibetan goats also indicated that RES reduces the ruminal abundance of *Aspergillus*, suggesting a role in maintaining microbial homeostasis and host health (Zhu et al., 2024). Furthermore, RES significantly influences the rumen microbiota composition and fermentation parameters (He et al., 2022). In Tibetan sheep, RES supplementation increased the ruminal abundances of *Fibrobacter succinogenes*, *Ruminococcus albus*, and *Butyrivibrio fibrisolvens* (Ma et al., 2015). These findings collectively highlight the potential of RES as a modulator of rumen microbial ecology in ruminants.

Although previous research has revealed RES-induced shifts in the rumen microbiota of Tibetan sheep (Zhu et al., 2024), systematic investigations into its effects on rumen histomorphology and key fermentation parameters remain limited. Therefore, this study aims to evaluate the impact of dietary RES supplementation on rumen histomorphology and fermentation parameters in Tibetan sheep. The goal is to elucidate its potential mechanisms in promoting rumen health and to provide a theoretical foundation for future applications aimed at enhancing production performance.

## 2 Materials and methods

The animal care and experimental protocols used were approved by the Institutional Animal Care and Use Committee of Qinghai University, China. (Approval number: QUA-2020-0710).

### 2.1 Experimental design and feeding management

A total of 120 healthy, 2-month-old weaned male Tibetan lambs (17.85 ± 0.5 kg) were randomly assigned to four dietary treatments: CK (basal diet), L-RES (basal diet + 1 g RES/d), M-RES (basal diet + 1.25 g RES/d), and H-RES (basal diet + 1.5 g RES/d). The 100-day trial comprised a 10-day adaptation period followed by a 90-day experimental period. The diets contained roughage (oat green hay and oat silage, 1:1 DM basis) and were concentrated at a 70:30 ratio (detailed formulation in Table 1). Sheep were housed at the Jinzang Ranch (Haiyan County, Haibei Prefecture, Qinghai Province, China) in a sanitized, temperature-controlled facility with adequate lighting, ventilation, and access to an exercise yard. During the preparation of the experimental feeds, resveratrol was added to the premix in advance, mixed thoroughly in a blender, and fed to each individual twice a day, at 8:00 and 17:00. The remaining feed was checked before each feeding to ensure that it was completely consumed. At the end of the feeding experiment, six Tibetan sheep were randomly selected from each group for slaughter.

**Table 1:** Composition and nutrient levels of the base ration.

Ingredient	Content
Corn	58.30
Soybean meal	1.00
Rapeseed meal	7.00
Cottonseed meal	2.00
Palm meal	25.00
NaCl	1.00
Limestone	1.00
Baking soda	0.10
Premix^1^	4.60
Total	100.00
Nutrient levels^2^	
DE/(MJ·kg^−1^)	12.84
Crude protein	12.13
Ether extract	3.44
Crude fiber	11.05
Neutral detergent fiber	26.04
Acid detergent fiber	19.11
Ca	0.80
P	0.40

^1^Premix provides 18 mg of Cu, 66 mg of Fe, 30 mg of Zn, 48 mg of Mn, 0.36 mg of Se, 0.6 mg of I, 0.24 mg of Co, 24,000 IU of VA, 4,800 IU of VD, and 48 IU of VE per kg of diet. ^2^Digestive energies are calculated values, and the rest are measured values.

### 2.2 Slaughtering and sample collection

At the conclusion of the trial, all Tibetan sheep (*n* = 6 per treatment) were slaughtered in a commercial slaughterhouse. Rumen fluid samples were subsequently collected, filtered through four layers of cotton cloth, and stored at −80 °C for future analysis.

### 2.3 Rumen tissue morphology

Rumen tissue samples fixed in 4% paraformaldehyde were processed, subjected to graded ethanol dehydration, xylene clearing, and paraffin embedding. Serial sections (5 μm thick) were mounted on glass slides, dried, and stained with hematoxylin and eosin (H&E). Morphometric analysis of ruminal papilla length, papilla width, and cuticle thickness was conducted via light microscopy with image analysis software.

### 2.4 Rumen fermentation parameters

The rumen fluid pH was measured immediately after collection via a calibrated portable pH meter (Leici PH-25-3C-3E, Shanghai, China). For volatile fatty acid (VFA) quantification, the rumen fluid was filtered through four layers of cheesecloth, preserved with 25% metaphosphoric acid (5:1 v/v), and centrifuged at 10,000 × *g* for 10 min. The supernatants were analyzed via gas chromatography (GC-2010, Agilent Technologies, Japan) equipped with a DB-FFAP capillary column (30 m × 0.32 mm × 0.25 μm), flame ionization detector, and helium carrier gas (1.0 mL/min). The temperature program commenced at 100 °C (2 min hold) with a 10 °C/min ramp to 240 °C.

### 2.5 Microbial 16S rRNA sequencing

Total genomic DNA was extracted from the rumen contents via the QIAamp Fast DNA Stool Mini Kit (Qiagen, Germany). The V3–V4 (2 min at 95 °C, then 27 cycles of 10 s at 98 °C, 30 s at 62 °C, 30 s at 68 °C, and finally 10 min of extension at 68 °C) region of the 16S rRNA gene was amplified with the universal primers 341F (5′-CCTACGGGNGGCWGCAG-3′) and 806R (5′-GGACTACHVGGGTATCTAAT-3′). The amplification products were verified via 2% agarose gel electrophoresis, purified via the AxyPrep DNA Gel Extraction Kit (Axygen Biosciences, United States), and quantified via a Qubit 2.0 fluorometer (Thermo Fisher Scientific, United States). Constructed libraries were quantified by NEB NextUltra™ DNA Library Prep Kit for Illumina (Diego, CA, USA) and indexing codes added. Purified amplicons were pooled in equimolar concentrations and sequenced on an Illumina HiSeq 2,500 platform to generate 300-bp paired-end reads. Sequences were clipped from their original labels using FLASH software (version 0.18.0). Valid labels were clustered into out-of-taxonomy units (OTUs) with ≥97% similarity using the Uparse pipeline (Gai et al., 2022). Calculate beta distances using the R package Vegan (version 2.5.3). Evaluating differences in the Bray–Curtis distance matrix using the R “ggplot2” package (version 2.2.1). Alpha diversity analysis was calculated in QIIME (version 1.9.1). Correlation analyses were analyzed on the Kidio Run platform.

### 2.6 Analysis of rumen metabolomics data

After the samples were thawed, an appropriate amount of the samples was added to a precooled methanol/acetonitrile/water solution (2:2:1, v/v), vortexed and mixed, and sonicated at a low temperature, and the supernatant was vacuum dried; for mass spectrometry, 100 μL of acetonitrile aqueous solution (acetonitrile: water = 1:1, v/v) was added to redissolve the samples, vortexed, and centrifuged at 14,000 × g at 4 °C for 15 min, after which the supernatant was injected into the sample for analysis. The samples were separated on an Agilent 1,290 Infinity LC ultrahigh-performance liquid chromatography (UHPLC) HILIC column. The primary and secondary spectra of the samples were acquired with an AB Triple TOF 6600 mass spectrometer.

The raw data were converted to the MzML format via ProteoWizard, and the XCMS program was subsequently used for peak alignment, retention time correction and peak area extraction. The data were imported into SIMCA-P 14.1 (Umetrics, Sweden) and subjected to orthogonal partial least squares discriminant analysis (OPLS-DA), with screening criteria for differentially abundant metabolites (DMs): variable importance projection (VIP) > 1 and *p* < 0.05 (t test). Metabolite functions were then annotated via the KEGG database^1^; pathway enrichment analysis was performed via MetaboAnalyst 5.0^2^, with the significance threshold set at FDR < 0.05.

### 2.7 Correlation analysis

Spearman correlation analysis was used to assess the relationships among the rumen microbiota, metabolites, histological morphology, and fermentation parameters. Statistical analyses were performed with the general linear model (GLM) in Origin Pro 2023, with the results expressed as the mean ± standard error of the mean (SEM). Statistical significance was defined as *p* < 0.05.

### 2.8 Statistical analysis of data

Experimental data were organized using Excel 2019 (Microsoft Corporation, United States) and statistically analyzed by SPSS 26.0 (IBM Corporation, United States). Since microbial community data usually did not satisfy normal distribution, non-parametric Kruskal–Wallis *H*-test was used for comparison between groups. *Post hoc* multiple comparisons were performed using Dunn’s test if the test was statistically significant (*p* < 0.05). Continuous data that conformed to a normal distribution were analyzed by one-way ANOVA with *post hoc* multiple comparisons combined with Duncan’s method. All results are presented as mean ± standard deviation (SD).

## 3 Results

### 3.1 Effect of dietary resveratrol addition on rumen morphology in Tibetan sheep

**Figure 1:**
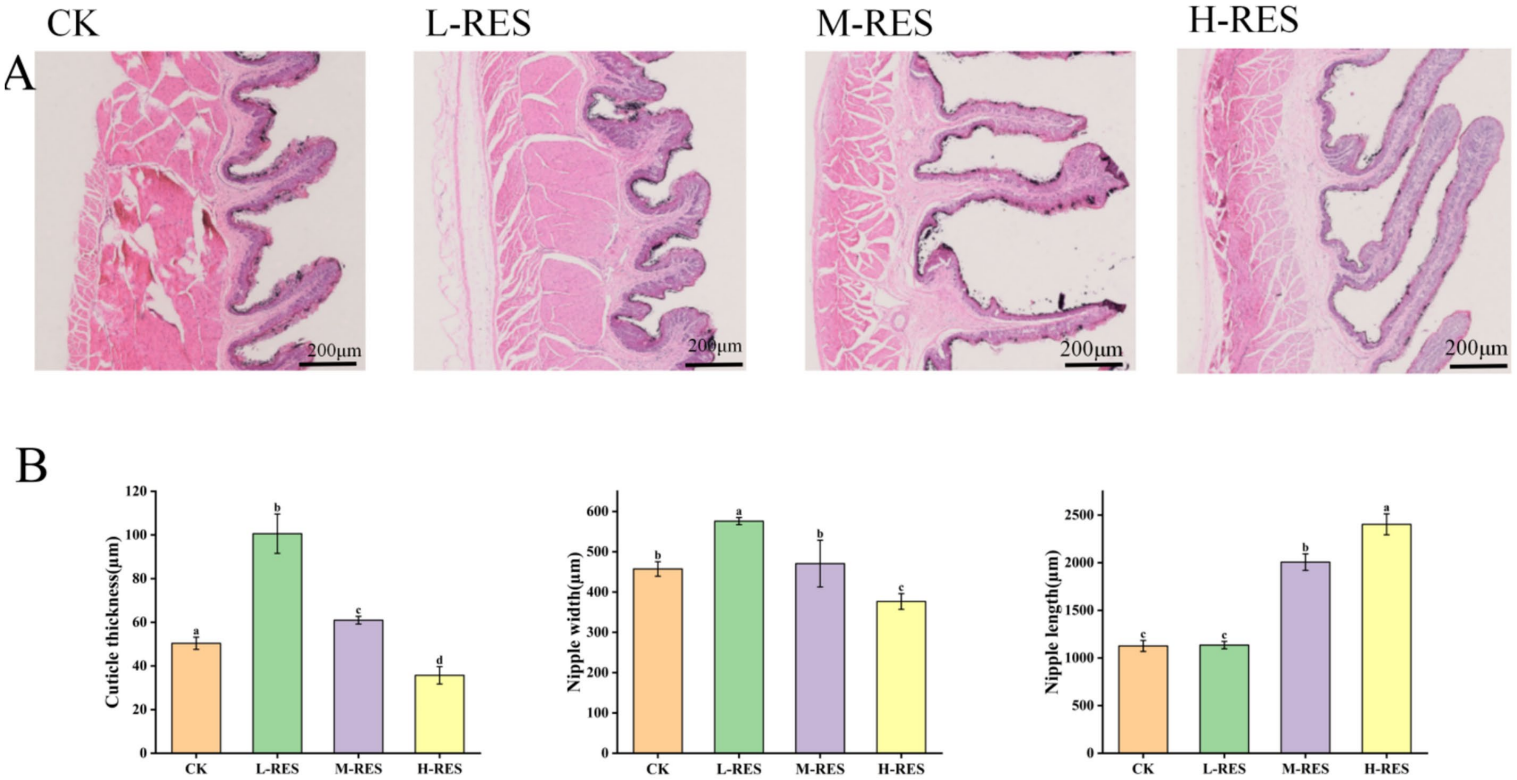
Effects of dietary supplementation with different doses of RES on rumen morphology. **(A)** Representative histological images of rumen sections after hematoxylin–eosin staining (original magnification 200 × μm). **(B)** Ruminal cuticle thickness, nipple width and nipple length.

Figure 1 shows dose-dependent alterations in ruminal papilla morphology among the RES-supplemented groups (*p* < 0.05). Compared with the other treatments, the H-RES group presented significantly longer papillae (*p* < 0.05), potentially increasing the ruminal epithelial surface area and nutrient absorption efficiency. Among them, the changes in rumen cuticle thickness were significant in the L-RES group compared with the CK group (*p* < 0.05). Collectively, RES supplementation modulated rumen morphogenesis in a dose-dependent manner, conferring protective effects on ruminal epithelial integrity. Meanwhile, the total VFA in the H-RES group were significantly higher than those in the CK group, indicating that the addition of H-RES significantly promoted the fermentation activity of microorganisms in the rumen.

### 3.2 Fermentation parameter results

As shown in Table 2, different RES additions significantly affected the rumen pH. The pH values of the H-RES group were significantly greater than those of the CK group. In contrast, the pH values of the M-RES group were significantly lower. Additionally, the acetic and butyric acid contents in the L-RES and H-RES groups were greater than those in the CK group (*p* < 0.05).

**Table 2:** Rumen fermentation parameters.

Fermentation parameters	CK	L-RES	M-RES	H-RES	*P*
pH	5.7333 ± 0.0022^c^	5.923 ± 0.013^b^	5.9200 ± 0.011^b^	5.8200 ± 0.011^a^	0.000
Acetic acid (mmol/L)	0.5605 ± 0.072^b^	0.7451 ± 0.012^b^	0.5558 ± 0.032^b^	0.6232 ± 0.052^a^	0.007
Propanoic acid (mmol/L)	0.0325 ± 0.014	0.0507 ± 0.012	0.0228 ± 0.002	0.0115 ± 0.002	0.085
Butyric acid (mmol/L)	0.0329 ± 0.012^b^	0.0347 ± 0.013^b^	0.0265 ± 0.004^b^	0.0569 ± 0.004^a^	0.035
Isovaleric acid (mmol/L)	0.0102 ± 0.004	0.0071 ± 0.003	0.0064 ± 0.001	0.0055 ± 0.003	0.181
Isobutyric Acid (mmol/L)	0.0102 ± 0.002^a^	0.0071 ± 0.001^ab^	0.0033 ± 0.003^b^	0.0034 ± 0.003^b^	0.044
Octanoic acid (mmol/L)	0.0035 ± 0.001	0.0036 ± 0.004	0.0034 ± 0.004	0.0037 ± 0.002	0.766
Heptanoic acid (mmol/L)	0.0023 ± 0.002	0.0021 ± 0.003	0.0020 ± 0.003	0.0021 ± 0.001	0.154
Volatile Fatty Acid	0.0210 ± 0.001	0.2466 ± 0.001	0.0125 ± 0.002	0.0310 ± 0.003	0.000

In the same row, values with different small letter superscripts are significantly different (*P* < 0.05), whereas values with the same or no letter superscripts are not significantly different (*p* > 0.05).

### 3.3 Differences in the diversity of rumen microbial communities in Tibetan sheep

**Figure 2:**
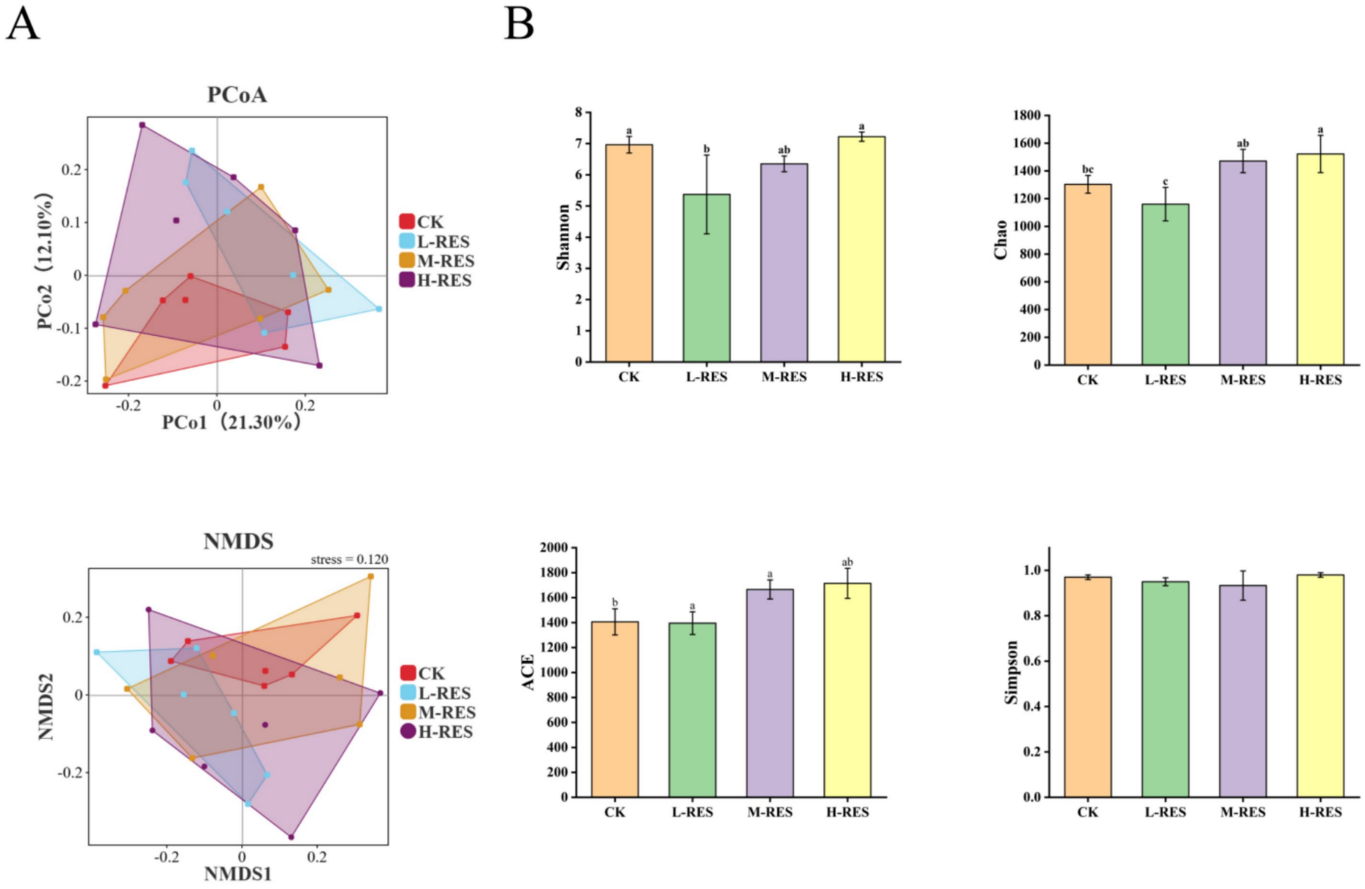
Effects of RES supplementation at different doses on rumen bacterial communities. **(A)** *α* diversity analysis. **(B)** β diversity analysis, *p* < 0.05. CK (basal diet), L-RES (basal diet + 1 g RES/d), M-RES (basal diet + 1.25 g RES/d), and H-RES (basal diet + 1.5 g RES/d).

The Venn diagram represents different species by different color blocks, and if the blocks overlap, it represents the number of shared species. A total of 1,693 operational taxonomic units (OTUs) were identified in rumen fluid samples from different RES treatments, of which 812 were shared OTUs (Supplementary Figure S1). Ruminal microbial community β-diversity was assessed by principal coordinate analysis (PCoA), which showed good reproducibility of samples within groups but extensive mixing of sample points between groups, with no clear trend of segregation observed (Figure 2). This was further supported by ANOSIM analysis, which revealed an R-value of 0.0076 and a *p*-value of 0.402, indicating no significant difference in the overall microbial community structure between the groups.

### 3.4 Effects of different dosage groups of RES on rumen microflora of Tibetan sheep

**Figure 3:**
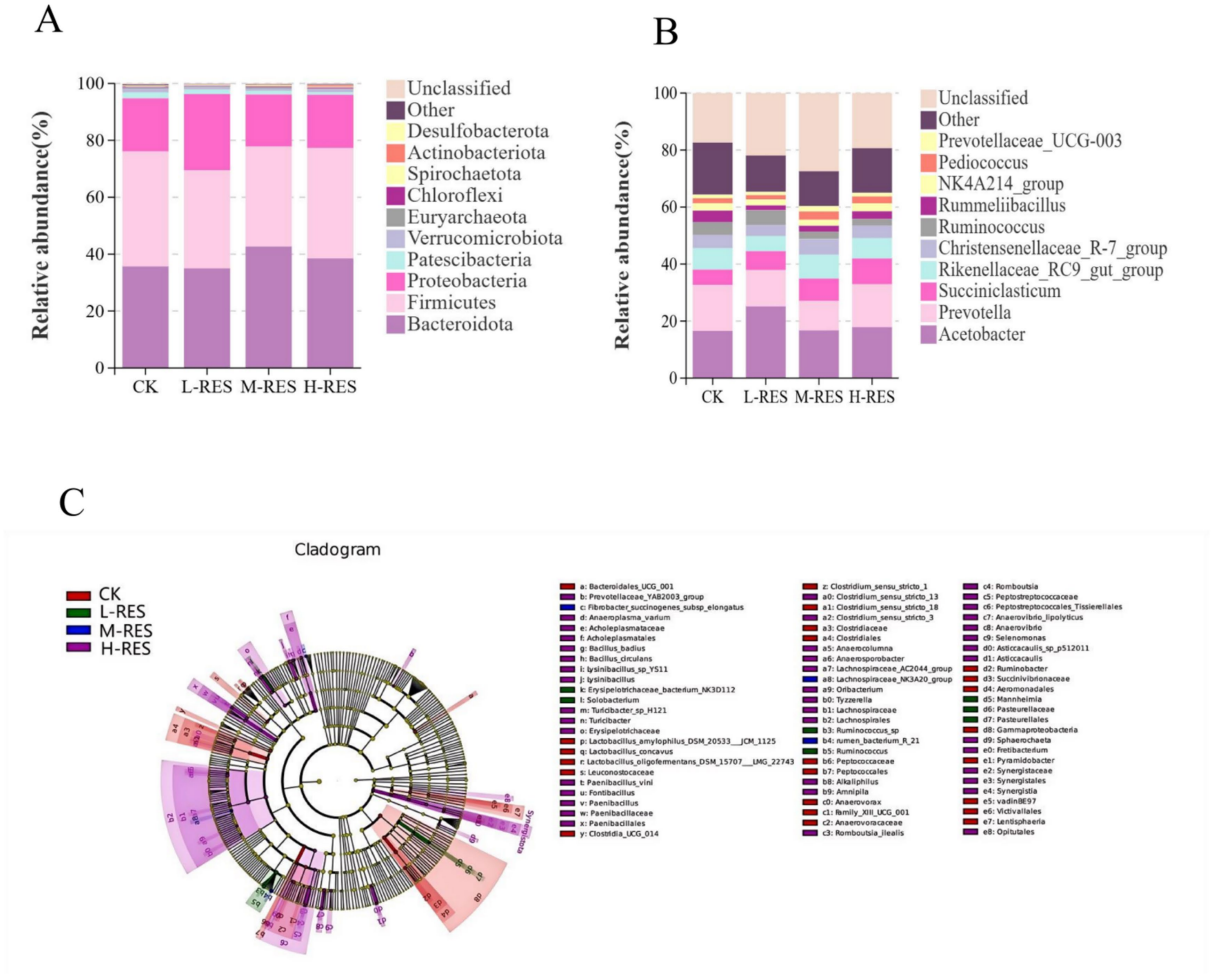
Effects of supplementation with different RES on bacterial composition. **(A)** Significant enrichment of rumen microbes at the phylum level. **(B)** Significant enrichment of rumen microbes at the genus level. **(C)** Lefse analysis of the four RES dose groups.

We selected the top 10 phyla and genera ranked by average relative abundance for in-depth analysis to further investigate the effects of RES on microbial communities. At the phylum level (Figure 3), Firmicutes and Bacteroidetes dominated the rumen microbiota composition, collectively accounting for >80% of the sequences, followed by Proteobacteria, Patescibacteria, Verrucomicrobiota, Euryarchaeota, Chloroflexi, Spirochaetota, Actinobacteriota, and Desulfobacterota, with subsequent relative abundances. Bacteroidetes levels were elevated in both the M-RES and H-RES groups compared to the CK group, while the L-RES group exhibited a slight decrease. Regarding Firmicutes, the L-RES group showed a significant reduction relative to the control group, whereas the M-RES and H-RES groups demonstrated only marginal increases compared to CK, with changes being non-significant.

The top 10 bacterial genera were detected at the genus level (Figure 3), of which *Acetobacter* and *Prevotella* were the two dominant genera in the intestinal rumen fluid of Tibetan sheep, followed by *Succiniclasticum*, the *Rikenellaceae RC9 gut group*, the *Christensenellaceae R-7 group*, *Ruminococcus*, *Rummeliibacillus*, the *NK4A214 group*, *Pediococcus*, and *Prevotellaceae UCG-003*. Among these dominant genera, 11 genera that varied in abundance at different RES levels were selected. Acetobacter abundance was significantly higher in the L-RES, M-RES, and H-RES groups compared to the CK group. Succiniclasticum abundance was lower in the CK group than in the other groups, while Prevotella abundance showed a decreasing trend in the remaining groups compared to the CK group (Supplementary Figure S2).

Lefse analysis of the four RES dose groups (Figure 3), with LDA = 3 as the reference value, revealed that there were significant differences in the rumen microbial species among the four groups of Tibetan sheep. At the phylum level, Proteobacteria were significantly enriched in the CK group. At the genus level, distinct marker genera were enriched across different dose groups: characteristic genera in the CK group included *Clostridium*, *Ruminococcus*, and *Clostridium sensu stricto 1*; within the L-RES group, *Ruminococcus* sp. showed significant enrichment; the M-RES group exhibited characteristic genera such as *Lachnospiraceae_nk3a20_group*; and the H-RES group showed the most abundant significantly enriched genera, including *Spirochaeta*, *Staphylospora*, *Anaerovibrio*, and *Anaerovibrio lipolyticus*. These findings indicate that supplementation with different doses of RES may induce significant differences in the rumen microbiota of Tibetan sheep.

### 3.5 Comparative study of the effects of different resveratrol concentrations on the rumen metabolism of Tibetan sheep experiment

**Figure 4:**
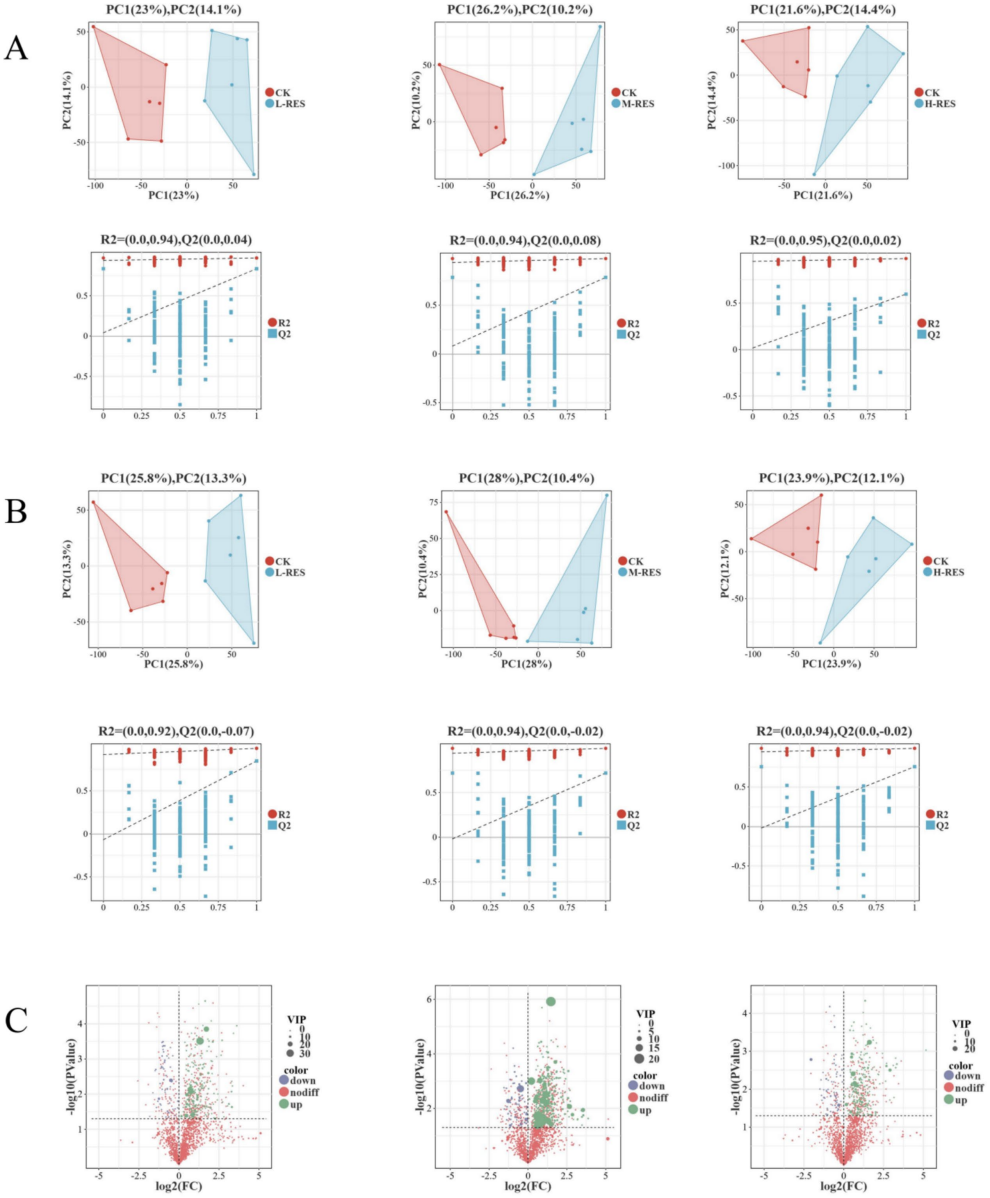
Comparison of orthogonal partial least squares discriminant analysis (OPLS-DA) and PCA plots of metabolites. **(A)** Positive ion mode. **(B)** Negative ion mode. **(C)** Volcano plot analysis between different dose groups.

Analysis of metabolic data from Tibetan sheep rumen (Figure 4) revealed robust intra-group clustering in both positive and negative ion modes for experimental samples, alongside distinct inter-group separation indicating treatment-related differences. Principal Component Analysis (PCA) assessment of sample group separation showed no significant outliers, confirming high data quality. To clearly evaluate treatment-induced metabolic differences between groups, supervised orthogonal partial least squares discriminant analysis (OPLS-DA) was employed. As shown in Figure 4, further assessment of the OPLS-DA model revealed significant differences in metabolite composition across groups in both positive and negative ion modes, indicating distinct metabolites obtained from rumen fluid samples at different RES doses. However, validation revealed limited predictive capability of the model. Subsequently, variable importance projections (VIP > 1.0) and univariate analysis (*p* < 0.05) were employed to screen reliable metabolites exhibiting significant intergroup differences and high contribution for further analysis. Based on LC–MS/MS analysis, a total of 2002 (positive ion mode) and 1,395 (negative ion mode) valid peaks were detected.

Basic analysis of rumen metabolic differences in Tibetan sheep revealed that according to the volcano plot analysis (Figure 4), there were 201 differentially abundant metabolites in the low-dose RES group compared with the control group, of which 170 were upregulated and 31 were downregulated; 233 differentially abundant metabolites in the medium-dose RES group compared with the control group, of which 201 were upregulated and 32 were downregulated; and 201 differentially abundant metabolites, of which 172 were upregulated and 29 were downregulated. Metabolic pathway enrichment analysis was performed to determine the number and significance level of pathways and the functions of different metabolites, and the following significantly differentially abundant metabolites were screened in the low- and medium-dose groups using VIP values >1 and *p* < 0.05 as reference thresholds. The following metabolites exhibiting significant differences were identified across the low-, medium-, and high-dose RES groups (Supplementary Table S1).

By KEGG biological pathway enrichment analysis, FDR < 0.05 signaling pathways were screened for enrichment analysis according to the DM, and after significance histogram analysis (S3), the metabolic pathways included mainly amino acid metabolic pathways, carbohydrate metabolic pathways, nucleotide metabolism, lipid metabolism, glycine, serine, and methionine metabolic pathways, pyrimidine metabolism, and microbes in different environmental metabolic pathways and other signaling pathways and fields but were not limited to the above pathways and fields. The signaling pathways varied in the different groups due to differentially abundant metabolites, and in the L-RES group, they were enriched mainly in metabolic pathways and glycine, serine, and methionine metabolic pathways. In the M-RES group, the major enrichment was in the metabolic pathway and the ABC transporter protein metabolic pathway. In the H-RES group, the amino acid metabolic pathway was the main pathway enriched. Compared with the individual analyses, the overall generational pathway was enriched mainly in the amino acid metabolic pathway.

### 3.6 Correlation analysis

**Figure 5:**
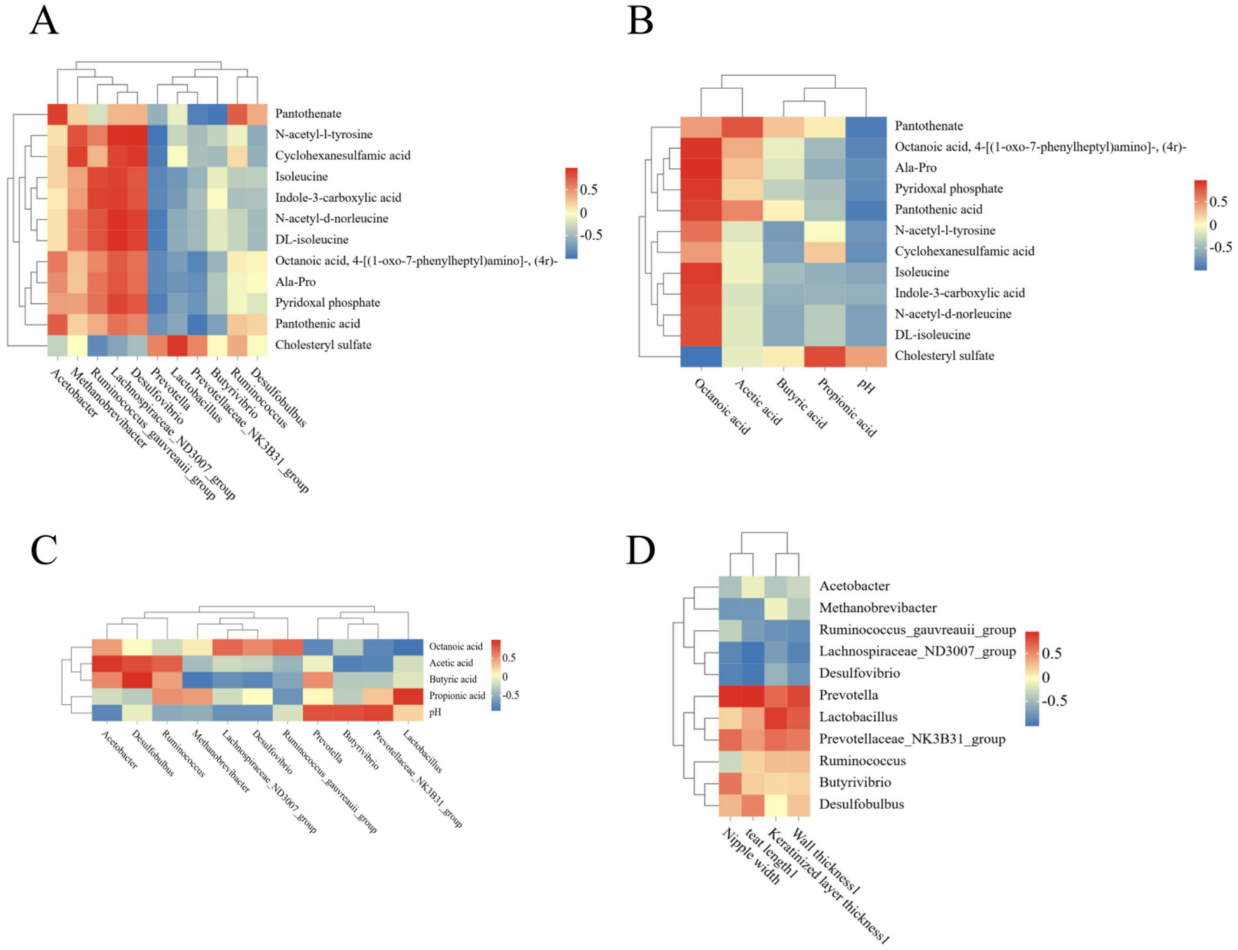
Correlation analysis between microorganisms, metabolites, ruminal fermentation parameters and rumen morphology. **(A)** Correlation analysis between rumen fermentation and metabolites. **(B)** Correlation analysis between metabolites and rumen fermentation parameters. **(C)** Correlation analysis between rumen fermentation parameters and microorganisms. **(D)** Correlation analysis between microorganisms and Rumen morphology. The color gradient represents the correlation coefficient (from blue for negative to red for positive). Cells are filled with color only for statistically significant correlations (*p* ≤ 0.05).

To identify metabolites associated with rumen fermentation, Pearson correlation analyses were performed on rumen fermentation indicators, rumen morphology, and differentially abundant metabolites in the filtrate (Figure 5). Cyclohexylsulfamic acid was negatively correlated with octanoic acid, and all other metabolites were correlated with octanoic acid. The correlation of microorganisms with rumen fermentation parameters revealed that cyclohexylsulfamate was negatively correlated with pH, whereas all other microorganisms were negatively correlated with pH. The correlation heatmap is shown in Figure 5D. *Lactobacillus* was strongly positively correlated with cholesterol sulfate, *Desulfovibrio*, *Lachnospiraceae_ND3007_group*, *Ruminococcus_gauvreauii_group*, *Lactobacillus* and acetic acid bacteria were strongly positively correlated with pantothenic acid, and N-acetyl-d-norleucine, N-acetyl-l-tyrosine, indole-3-carboxylic acid, DL-isoleucine, octanoic acid, 4-[(1-oxo-7-phenylheptyl) amino]-, (4r)-, alanyl-propyl, cyclohexylamino- sulfonic acid, pantothenic acid and pyridoxal phosphate were positively correlated with pantothenic acid.

## 4 Discussion

Resveratrol (RES), a natural polyphenol, has been demonstrated to modify the rumen environment in Tibetan sheep (Gan et al., 2024). It functions by selectively modulating the microbial community structure, thereby altering rumen fermentation parameters and promoting rumen morphogenesis. This suggests a potential mechanism through which RES regulates rumen function in ruminants.

The rumen, as the primary digestive organ of ruminants, relies on stability of its microbial environment for efficient digestion and absorption. Alterations in the microbial community structure can compromise the homeostasis and development of the rumen (Jami et al., 2013). This study found that RES supplementation significantly modulated rumen microbial *α*-diversity, with the H-RES group showing a significant advantage over L-RES and M-RES groups in both the ACE index (*p* < 0.05) and Shannon index (*p* < 0.05). This observation aligns with previous findings on microbial species richness and diversity (Shen et al., 2022). The observed microbial shifts suggest an optimization of carbohydrate and nitrogen metabolism. The phylum Bacteroidetes, a major source of carbohydrate-active enzymes (CAZymes) (Wardman et al., 2022), is crucial for the degradation of cellulose and hemicellulose (Naas et al., 2014). Enhanced degradation of hemicellulose, a primary constituent of plant cell walls (Scheller and Ulvskov, 2010), is a key indicator of improved feed efficiency (Weimer, 2022). Consistent with this, the alterations in microbial structure in the H-RES group correlated with a significant increase in ruminal VFA concentrations. This suggests that RES optimizes the microbial community to enhance the metabolism of coarse fodder, thereby increasing energy (acetate, propionate, and butyrate) supply to the host and promoting rumen development. Butyrate is particularly significant, as it serves as the primary energy source and proliferative signal for rumen epithelial cells, directly promoting papilla development (Sun et al., 2018). *Prevotella* are closely associated with amino acid metabolism and protein degradation (Patra and Yu, 2022), and by secreting peptidases, they synthesize amino acid precursors to participate in host metabolic processes. Furthermore, under anaerobic conditions, they hydrolyze dietary proteins into free amino acids and undergo deamination via the glutamate dehydrogenase pathway to produce short-chain fatty acids, thereby supplying energy to the host (Betancur-Murillo et al., 2022). Changes in *Prevotell*a abundance indicate alterations in ruminal nitrogen metabolism (Wang Y. et al., 2020), which is consistent with the significant upregulation of N-acetyl tyrosine observed in the M-RES group in our metabolomics analysis. In this study, the relative abundance of *Lactobacillus* in the rumen was significantly reduced in all treatment groups compared with the CK group. In terms of nitrogen metabolism, the high-dose treatment groups showed a significant reduction in Lactobacillus abundance compared to the CK group, which corresponded with a significant elevation in rumen pH. This collective evidence indicates that RES effectively mitigates the risk of acidosis by preventing lactic acid accumulation, thus maintaining a stable rumen environment (Jaramillo-López et al., 2017). Furthermore, the core fiber-degrading genera, including *Ruminococcus* and *Butyrivibrio* (Ilina et al., 2018), were also significantly affected. Notably, *Butyrivibrio* is a key butyrate producer, and its increased abundance was consistent with the significantly elevated butyrate concentration in the H-RES group (Palevich et al., 2024). In this study, the H-RES group exhibited significantly elevated butyrate concentrations, consistent with increased *Butyrivibrio* abundance. Correlation analysis confirmed a significant positive relationship between *Butyrivibrio* relative abundance and rumen papilla length. It is suggested that RES treatment may influence rumen fermentation patterns and promote rumen morphology development by altering microbial community structure. Finally, the observed community changes point toward enhanced energy utilization efficiency. The increased abundance of *Methanobrevibacter* suggests a potential reduction in ruminal hydrogen partial pressure, which would facilitate NADH reoxidation and the production of efficient energy-yielding fatty acids (Ungerfeld, 2020). Although hydrogen partial pressure was not measured, the corresponding increase in total VFA levels in the H-RES group supports this inference. Concurrently, the decline in *Desulfovibrio* abundance suggests a reduction in the production of toxic by-products, further optimizing the fermentation process (Yan et al., 2019). These integrated alterations in microbial structure underpin the observed changes in rumen metabolism.

Alterations in the rumen microbial structure of Tibetan sheep through ration supplementation with RES provided the basis for changes in rumen metabolism and were reflected in enhanced microbial anabolism. Pyridoxal phosphate is involved in amino acid metabolism and is a coenzyme in more than 140 enzymatic reactions (Gandhi, 2009), and can participate in transamination, decarboxylation, cleavage, and other reactions for large-scale amino acid conversions (Frese, 2018). Concurrently, pantothenic acid (vitamin B5) is a precursor for acetyl coenzyme A (CoA) (Leonardi and Jackowski, 2007), which is critical for VFA production, such as acetic acid (Miller and Rucker, 2020). These fatty acids in turn are extensively involved in the body’s sugar, lipid (Chandel, 2021), and amino acid metabolism (Kalhan, 2009). The significant upregulation of both pyridoxal phosphate and pantothenic acid across the RES treatment groups, coupled with a positive correlation between pantothenic acid and acetic acid concentrations, leads to the hypothesis that RES stimulates acetic acid production by modulating pantothenic acid metabolism. The synergistic up-regulation of these two cofactors suggests a coordinated metabolic response that enhances energy metabolism and short-chain fatty acid production.

The amino acid metabolic pathway becomes abnormally active, driven by a variety of enzymes such as pyridoxal phosphate (Wakita and Hoshino, 1975), which was manifested in this study by the accumulation of a variety of metabolites. Isoleucine upregulation, where the rate of degradation of endogenous proteins exceeds the rate at which microorganisms use them to synthesize their own proteins (López-Hernández et al., 2023), may be directly related to the enrichment of protein-degrading bacteria such as *Prevotella*. This counter-intuitive finding suggests that *Prevotella* enrichment did not cause amino acid accumulation but rather accelerated the overall amino acid turnover, with microorganisms rapidly converting protein degradation products into their own biomass, which ultimately contributes to increased total VFA concentrations (López-Hernández et al., 2023). The upregulation of the modified amino acid N-acetyl-d-norleucine further validates this accelerated amino acid metabolism, consistent with previous findings (Xu et al., 2024). Furthermore, Ala-Pro, a marker of protein hydrolysis, was significantly upregulated in rumen metabolites. This suggests that the rate of peptide production exceeded the rate of subsequent hydrolysis by peptidases or microbial uptake (Wallace et al., 1999). Correlation analysis revealed a significant synergism between protein degradation and carbohydrate fermentation, as Ala-Pro concentration was positively correlated with both acetic acid and Ruminococcus abundance. We hypothesize that the protein degradation represented by Ala-Pro releases oligopeptides and amino acids, providing a nitrogen source for fiber-degrading bacteria (e.g., *Ruminococcus*) (Argyle and Baldwin, 1989). This enhanced nitrogen supply, in turn, boosts the decomposition of cellulose by fiber-degrading bacteria, resulting in the simultaneous increase in acetic acid production, Finally, the upregulation of indole-3-carboxylic acid (a tryptophan metabolite) may reflect a diverted microbial metabolic pathway chosen in response to high amino acid metabolism, serving to maintain the stability of the ruminal environment (Esperanza et al., 2020).

The integration of shifts in microbial structure and metabolites ultimately drove changes in rumen fermentation end products. The accelerated amino acid degradation increased CoA availability, subsequently elevating the total VFA concentration in the H-RES group. Mechanistically, the increased acetyl-CoA from amino acid metabolism is known to promote butyric acid synthesis (Mousa et al., 2025). Supporting this, butyric acid concentrations were significantly higher in the H-RES group (*p* < 0.05), which coincided with significantly greater rumen papilla lengths. This confirms that RES improves microbial community function to enhance butyric acid production, which, as a key energy substrate and signaling molecule (Laarman et al., 2013), directly promotes rumen papilla development and optimizes absorption. This morphogenesis is further supported by specific microbial correlations: rumen papilla development was positively correlated with *Butyrivibrio* abundance (a butyrate producer), and negatively correlated with the methanogen *Methanobrevibacter*. The latter correlation is consistent with the premise that more efficient energy metabolism patterns favor rumen morphogenesis (Wang Q. et al., 2020). Collectively, these findings demonstrate that high-dose RES supplementation significantly contributes to a shift in the fermentation pattern toward butyric acid. This transformation not only optimizes the host’s energy metabolism efficiency but also directly stimulates rumen papillae development via butyric acid as a signaling molecule and energy substrate, thereby enhancing rumen absorptive capacity.

Through a multi-omics integration analysis, this study successfully elucidated the specific pathways by which RES regulates rumen function in Tibetan sheep, offering a robust theoretical basis for its application as a natural feed additive in ruminants. However, the study is subject to several limitations. While we established strong correlations between microorganisms and metabolites, the direct causal mechanisms remain partially unclarified. Specifically, the extent to which RES is degraded by rumen microorganisms and the activities of its resultant metabolites were not addressed. Future research should leverage metagenomics techniques and *in vitro* validation to fully clarify the causal relationships and the fate of RES in the rumen environment.

## 5 Conclusion

In this study, we investigated the effect of resveratrol on rumen function of Tibetan sheep by adding resveratrol to diets at different levels. The results showed that the high dose of RES significantly increased the rumen microbial *α*-diversity and changed the microbial community structure, as evidenced by a decrease in the abundance of *Lactobacillus* spp. and an increase in the abundance of beneficial bacteria such as *Vibrio butyricus* spp. These changes in microbial structure further induced changes in rumen fermentation parameters: rumen pH, total VFA, and butyric acid concentrations were significantly increased in the H-RES group. Correlation analysis showed a significant positive correlation between *Butyrivibrio* abundance and butyric acid concentration. Finally, the length and width of rumen papillae in the H-RES group were significantly greater than those in the other groups, and the morphological indicators were positively correlated with both butyric acid concentration and *Butyrivibrio* abundance. In conclusion, resveratrol can improve the rumen health of Tibetan sheep by regulating the structure and metabolic function of the rumen microbial community, optimizing the fermentation pattern, and promoting the development of rumen epithelium.

## Data Availability

The data presented in this study are publicly available. This data can be found at: https://www.cncb.ac.cn, accession number OMIX011289; https://www.ncbi.nlm.nih.gov, accession number PRJNA1299956.

## Footnotes

^1^ http://www.genome.jp/kegg/

^2^ https://www.metaboanalyst.ca/
