# Supplementary material for: Dietary resveratrol modulates rumen microbiota and metabolic function in Tibetan sheep: an integrated 16S rRNA sequencing and metabolomics analysis
Source: Front Microbiol. 2025 Dec 12;16:1679856. doi: 10.3389/fmicb.2025.1679856 (PMC12742471; doi:10.3389/fmicb.2025.1679856)
Supplement: Supplementary file 1 [file Supplementary_file_1.docx]

Supplementary Material

# Supplementary Figures and Tables


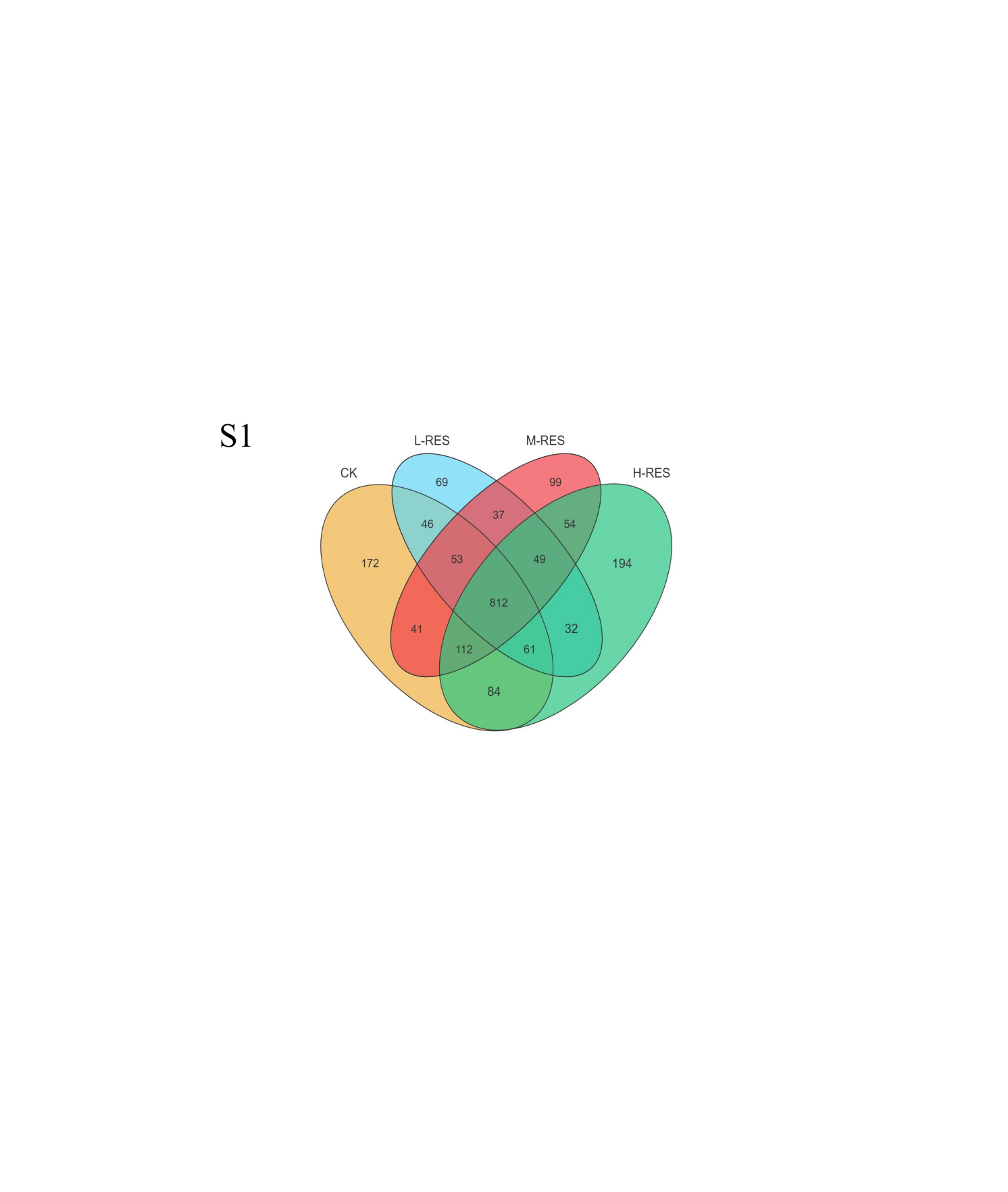


**Figure S1** The Venn diagram illustrates the distribution of specific and shared operational taxonomic units (OTUs) across different dosage groups (0 g RES/d, 1 g RES/d, 1.25 g RES/d, 1.5 g RES/d).


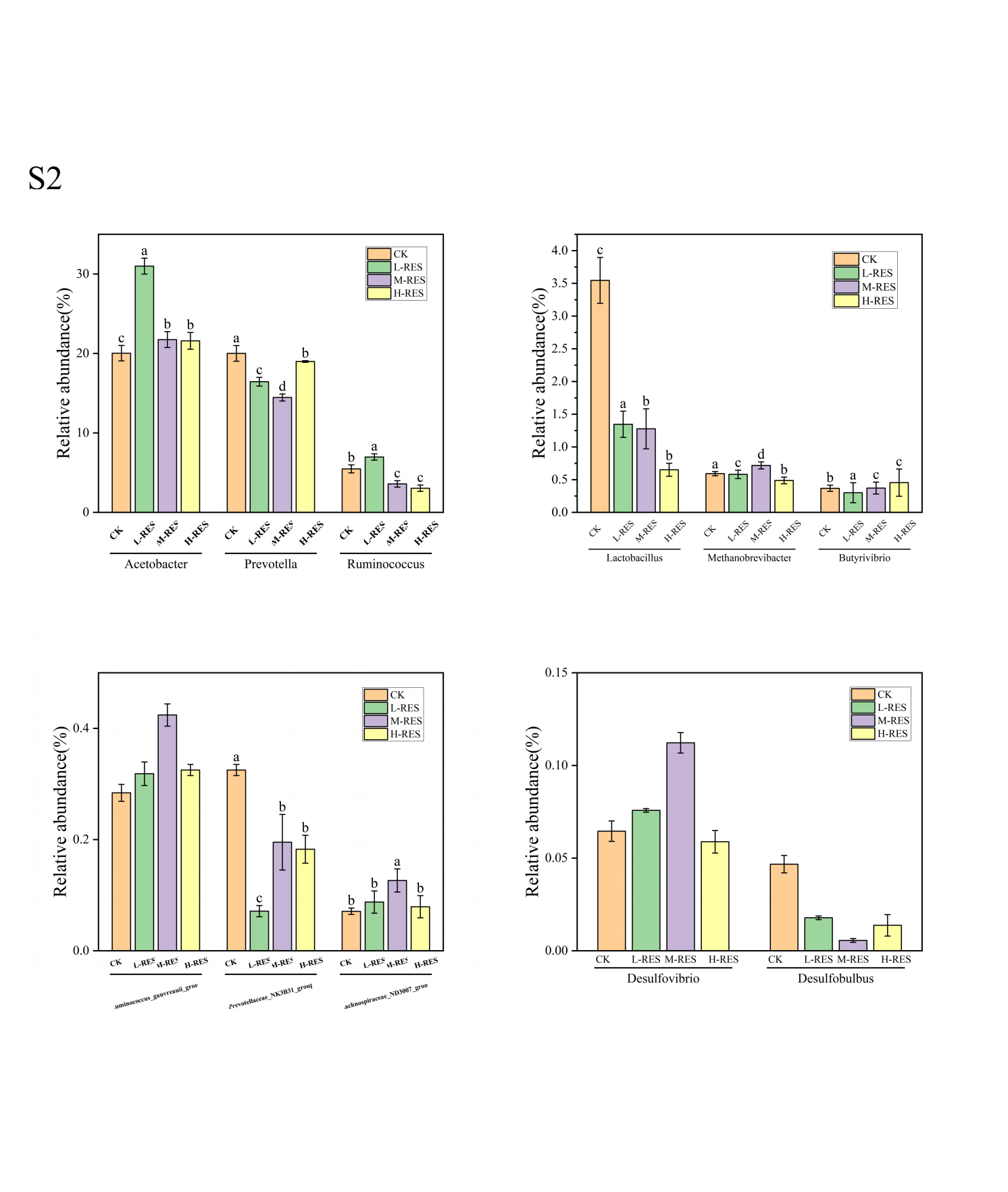


Figure S2 Dose-dependent shifts in the relative abundance of key microbial genera. Bar chart displays genera whose abundance was significantly altered by resveratrol supplementation (0 g RES/d, 1 g RES/d, 1.25 g RES/d, 1.5 g RES/d). Data are shown as mean ± SD. Bars with different letters (a, b, c) are significantly different from each other (*P* < 0.05).


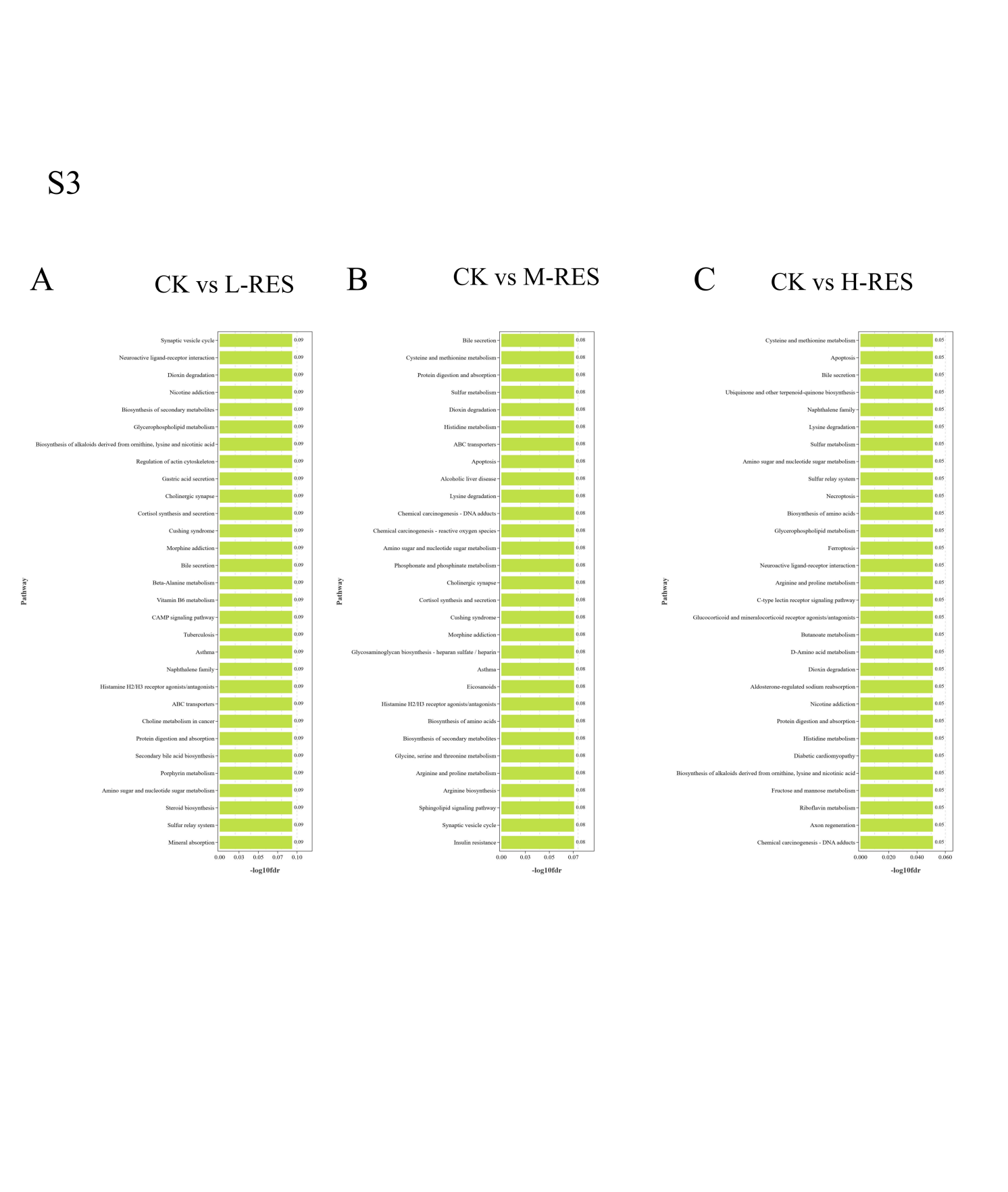


**Figure S3** KEGG pathway enrichment analysis reveals metabolic shifts in response to resveratrol. Significantly enriched pathways (*P* < 0.05) in the rumen microbiota of the RES-treated groups (1 g RES/d, 1.25 g RES/d, 1.5 g RES/d) compared to the control (CK) group are shown.

Table S1 Dose-dependent alterations in the rumen metabolome in response to resveratrol. Metabolites showing significant differences (*P* < 0.05) among the RES dose groups and the control (CK) group are displayed.

| Compounds | VIP | P-value | log2FC | fdr |
| --- | --- | --- | --- | --- |
| CK vs L-RES |  |  |  |  |
| Isoleucine | 11.57 | 0.02 | 0.90 | 0.13 |
| Indole-3-carboxylic acid | 2.38 | 0.02 | 0.46 | 0.11 |
| DL-isoleucine | 3.00 | 0.00 | 1.02 | 0.02 |
| Cholesteryl sulfate | 2.13 | 0.03 | -1.28 | 0.14 |
| Octanoic acid, 4-[(1-oxo-7-phenylheptyl) amino]-, (4r) | 1.99 | 0.00 | 1.34 | 0.02 |
| Ala-Pro | 1.47 | 0.00 | 1.51 | 0.08 |
| Cyclohexanesulfamic acid | 1.24 | 0.00 | 1.92 | 0.02 |
| Pantothenic acid | 1.41 | 0.00 | 1.39 | 0.05 |
| Pyridoxal phosphate | 1.32 | 0.00 | 0.98 | 0.01 |
| CK vs M-RES |  |  |  |  |
| Isoleucine | 16.98 | 0.03 | 1.12 | 0.04 |
| Pantothenate | 13.85 | 0.02 | 1.10 | 0.03 |
| N-acetyl-d-norleucine | 11.24 | 0.00 | 1.77 | 0.01 |
| Indole-3-carboxylic acid | 4.43 | 0.00 | 0.93 | 0.06 |
| DL-isoleucine | 4.57 | 0.00 | 1.63 | 0.02 |
| Cholesteryl sulfate | 2.85 | 0.00 | -2.09 | 0.06 |
| Octanoic acid, 4-[(1-oxo-7-phenylheptyl) amino]-, (4r) | 2.22 | 0.01 | 1.55 | 0.08 |
| Ala-Pro | 2.07 | 0.00 | 1.87 | 0.03 |
| Cyclohexanesulfamic acid | 1.33 | 0.02 | 2.32 | 0.12 |
| Pantothenic acid | 1.80 | 0.00 | 1.67 | 0.03 |
| Pyridoxal phosphate | 1.58 | 0.00 | 1.13 | 0.01 |
| CK vs H-RES |  |  |  |  |
| Isoleucine | 15.40 | 0.00 | 0.81 | 0.10 |
| N-acetyl-d-norleucine | 12.07 | 0.02 | 0.88 | 0.18 |
| Indole-3-carboxylic acid | 4.76 | 0.00 | 0.82 | 0.07 |
| DL-isoleucine | 4.54 | 0.00 | 1.33 | 0.04 |
| Cholesteryl sulfate | 2.84 | 0.01 | -1.55 | 0.14 |
| Octanoic acid, 4-[(1-oxo-7-phenylheptyl) amino]-, (4r) | 2.40 | 0.01 | 1.43 | 0.13 |
| Ala-Pro | 2.09 | 0.00 | 1.62 | 0.09 |
| Cyclohexanesulfamic acid | 1.75 | 0.00 | 2.27 | 0.09 |
| Pantothenic acid | 1.62 | 0.03 | 1.41 | 0.19 |
| Pyridoxal phosphate | 1.62 | 0.00 | 1.02 | 0.06 |
